# Recommendations for Physical Inactivity and Sedentary Behavior During the Coronavirus Disease (COVID-19) Pandemic

**DOI:** 10.3389/fpubh.2020.00199

**Published:** 2020-05-12

**Authors:** Fabrizio Ricci, Pascal Izzicupo, Federica Moscucci, Susanna Sciomer, Silvia Maffei, Angela Di Baldassarre, Anna Vittoria Mattioli, Sabina Gallina

**Affiliations:** ^1^Department of Neuroscience, Imaging and Clinical Sciences, “G. D'Annunzio” University, Chieti, Italy; ^2^Department of Clinical Sciences, Lund University, Malmö, Sweden; ^3^Department of Medicine and Aging Sciences, University “G. D'Annunzio” of Chieti-Pescara, Chieti, Italy; ^4^Department of Cardiovascular, Respiratory, Geriatric, Nephrology and Anesthesiology Sciences, Sapienza University of Rome, Rome, Italy; ^5^Cardiovascular and Gynaecological Endocrinology Unit, Fondazione G. Monasterio CNR-Regione Toscana, Pisa, Italy; ^6^Surgical, Medical and Dental Department of Morphological Sciences Related to Transplant, Oncology and Regenerative Medicine, University of Modena and Reggio Emilia, Modena, Italy

**Keywords:** physical activity, sedentary behavior, cardiovascular prevention, COVID 19, quarantine, coronavirus

Since the escalation of coronavirus disease 2019 (COVID-19) pandemic, over a billion people across the world have faced restrictions due to varying degrees of confinement, and in the absence of a vaccine against SARS-CoV-2, massive public health interventions have been implemented to contain the outbreak. The lockdown set up in many countries to combat the COVID-19 epidemic entails unprecedented disruption of lives and work, determining specific risks related to mental and physical health in the general population, especially among those who stopped working during the current outbreak (1). The implementation of confinement policies to contain COVID-19 could be a catalyst for concealed mental and physical health conditions, further enhancing the effects of psychosocial risk factors, including stress, social isolation, and negative emotions that may act as barriers against behavioral changes toward an active lifestyle and negatively impact on global health, well-being and quality of life, ultimately resulting in result in a range of chronic health conditions (2, 3).

## Hazards Related to Physical Inactivity and Sedentary Behavior

The World Health Organization (WHO) classified physical inactivity as the fourth leading risk factor accounting for 6% of global mortality, following hypertension (13%), smoking (9%) and diabetes (6%). The relationship between physical inactivity and obesity trends was quite evident since 1953 when the London Busmen Study showed that bus drivers who mainly sat during work presented with larger waist circumferences, higher levels of adiposity and increased risk of coronary events than bus conductors, who walked the aisles and climbed the stairs of double-decker buses (4).

Physical inactivity levels are rising in many countries with significant implications for the prevalence of non-communicable diseases and the general health of the population worldwide. The WHO recommends that adults accumulate at least 150 min of moderate to vigorous-intensity physical activity (MVPA) or 75 min of vigorous-intensity physical activity (VPA) throughout the week, cumulated in bouts lasting ≥10 min. This volume of physical activity (PA) is associated with a lower risk of cardiovascular (CV) morbidity and mortality and a number of other healthcare benefits (5). Unfortunately, attained levels of daily PA are largely insufficient, especially in western countries.

Recent evidence suggests that sedentary behavior (SB) is independently associated with traditional CV risk factors and increased CV morbidity and global mortality, regardless of PA volume (6). SB is defined as any waking behavior characterized by an energy expenditure ≤ 1.5 metabolic equivalents, while in a sitting, reclining or lying posture. Typical SB includes “screen time” (TV viewing, videogame playing, computer use), car-driving, and reading. Importantly, in a dose-response meta-analysis of 34 studies, including 1,331,468 community-dwelling participants, total sitting time volumes >8 h and 6 h/day were associated with increased risk of all-cause death and CV death, respectively, in PA adjusted analyses (7). For TV viewing time, an increased risk for all-cause and CV mortality was strongest above levels of 3–4 h/day, regardless of PA level (7).

Thus, physical inactivity and SB should be considered as separate entities with their unique determinants and health consequences, but with synergistic harmful effects on CV health (8).

While containing the spreading of the contagion as quickly as possible is the urgent public health priority, there have been few public health guidelines for the public as to what people can or should do in terms of maintaining their daily exercise or PA routines (9, 10). Safeguarding psycho-physical health in a lockdown situation is paramount, and special attention should be paid to elderly and pediatric populations. With advancing age, it becomes more difficult to reverse the effects of deconditioning of the musculoskeletal system. Children and adolescents have higher PA needs than adults, and these are more difficult to achieve during the quarantine period, also due to the influence of home environment (11). Both physical and social environmental factors operating within the home space are indeed important influences on SB and PA, especially for the pediatric population (12). Regarding adolescents, another point that warrants careful vigilance concerns the risks associated with increased total screen time, including the total hours spent on computer, TV and video gaming.

WHO just released guidance intended for people in self-quarantine without any symptoms or diagnosis of acute respiratory illness, containing a set of practical advice on how to stay active and reduce SB while at home. WHO further highlights how standard recommendations of 150 min of MVPA or 75 min of VPA per week, or a combination of both, can still be achieved even at home, with no special equipment and with limited space.

## Tips for Home-Based Physical Activity and Sedentary Behavior Interruption

There is a robust health rationale for staying active at home in the current precarious environment, for all age groups. The following are general recommendations, unless otherwise specified.

**Table:** 

Take active short breaks	You can meet weekly recommendations performing short bouts of PA, including taking the stairs, performing domestic chores, such as cleaning and gardening, or funniest activities such as dancing.
Walk and stand up	Take every chance to walk and stand up, like walking during a call, or taking a breath of fresh air, even just at the window. Try not to sit continuously for more than 1 h, but rather to take a 1–2 min break every 30 min. Alternatively, consider active breaks every 2 h of SB or distribute periods ≥10 min of continuous aerobic activity throughout the day. Light-intensity activities like mobilizing the muscular masses and the joints are fine. Older people can perform them even in sitting or semi-lying position.
Follow online exercise classes, play with children, help the elderlies to stay active	Take the advantage of free, virtual exercise classes on the web, devote more time to playing with children and encourage seniors to stay safe and active choosing suitable exercises for endurance, strength, balance, and flexibility. Avoid screen time while playing with children in favor of funny activities and active playing. For children and teens, it is advisable to play with sports or fitness video games with motion sensor controls. Performing light-intensity activities while assisting older people protects you from sedentariness. Active play rather than screen time helps you and your children to avoid snacking.
Be regular	Have regular times for main meals, sleep, and wake-up calls. Your sleep should be of sufficient duration and good quality. Prioritize continuity and regularity rather than the intensity of the PA and gradually increase frequency, duration, and intensity. Activity trackers and smartphone apps can help in monitoring your progress. In case of poor experience and poor physical fitness, be careful.

**Figure 1:**
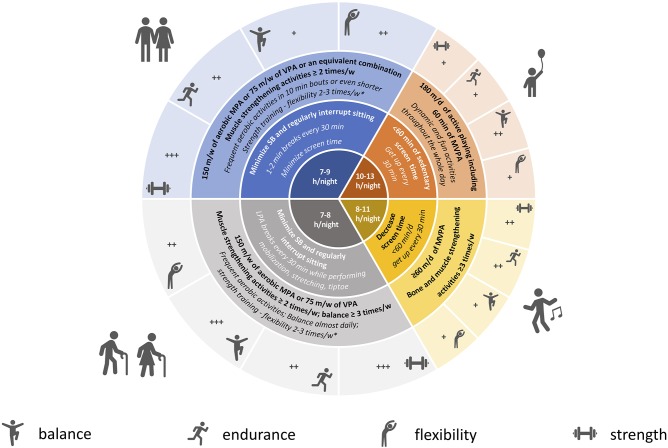
Physical activity, sedentary behavior, sleep recommendations, and tips for COVID-19 quarantine period. Blue, adults; gray, older people; orange, preschooler; yellow, school-aged children and adolescents; Bold, international guidelines and recommendations; Italic, tips for quarantine period; PA, physical activity; SB, sedentary behavior; LPA, light-intensity physical activity; MPA, moderate-intensity physical activity; VPA, vigorous-intensity physical activity; MVPA, moderate to vigorous-intensity physical activity. In the central portion of the figure we reported recommended hours of sleep by age group. ^*^Perform strengthening activities in non-consecutive days. +, ++, +++: relative importance of PA/exercise type for each age category. Dumbbell: muscle and bone strengthening activities; running: aerobic activities; monopodalic standing: balance exercise; bending: flexibility.

Specific recommendations and tips for children, adults, and elderly are further detailed in Figure 1.

## Conclusions

While recognizing the importance of confinement policies set up to contain COVID-19 pandemic, we firmly recommend the relevance of home-based programs for disruption physical inactivity and sedentary behavior as a critical behavioral strategy for the prevention of global health and consequences of psychosocial stress during the current lockdown.

## Author Contributions

FR and PI drafted the manuscript. All co-authors provided critical revision for important intellectual content.

## Conflict of Interest

The authors declare that the research was conducted in the absence of any commercial or financial relationships that could be construed as a potential conflict of interest.
